# Novel Coronavirus (COVID-19) Knowledge, Attitude, and Practice among Dental Sciences and Internship Students in Ajman University, United Arab Emirates: A Cross-Sectional Study

**DOI:** 10.1155/2023/3815286

**Published:** 2023-07-14

**Authors:** M. A. Jaber, Mohammed B Q Alfarra, Mawada Hassan Abdelmagied, Sudhir Varma, Essra El-Ameen, Salem Abu Fanas

**Affiliations:** ^1^Department of Clinical Sciences, College of Dentistry, Ajman University, Ajman, UAE; ^2^Center for Medical and Bio-Allied Health Sciences Research, Ajman University, Ajman, UAE; ^3^College of Dentistry, Ajman University, Ajman, UAE; ^4^College of Medicine, Ajman University, Ajman, UAE

## Abstract

**Background:**

The aim of this study was to assess the COVID-19-related knowledge, attitudes, and practice among dental students (DS) and internship students at Ajman University (AU).

**Methods:**

A cross-sectional, web-based study was conducted among Ajman dental college students registered in the academic year 2019/2020, about COVID-19 during the first week of April 2020. A questionnaire was developed and distributed to all DS to examine their knowledge and attitudes toward the COVID-19 pandemic. Chi-square (*χ*^2^) test was used to investigate the level of association among categorical variables at the significance level of *p* < 0.05.

**Results:**

Out of 769 students in the dental college, majority (75%) had adequate knowledge of COVID-19, but only 40.6% of the participants projected a positive attitude. A positive behavior was reported by an overwhelming 94.5% of the participants. Participants above 25 years of age had significantly higher (*p* < 0.001) adequate knowledge (88.2%), positive attitude (56.9%), and positive behavior (100%) compared to those below 25 years of age. Study participants reported social media (71.1%), government websites (63.7%), and family and friends (41.0%) as top three sources of information regarding COVID-19 related information.

**Conclusion:**

The results of this study show that AU dental and internship students have adequate knowledge and positive attitude of the COVID-19 pandemic but the majority are not eager to adopt effective strategies to avoid the spread of COVID-19 (practice). Further education should be offered to encourage efficient infection management practices to protect students, faculty, and other university staff.

## 1. Introduction

The first cases of novel coronavirus (nCoV) were first detected in China in December 2019, with the virus spreading rapidly to other countries across the world. This led WHO to declare a Public Health Emergency of International Concern on 30 January 2020, and to characterize the outbreak as a pandemic on March 11, 2020 [1]. This has led to challenges in medical treatment delivery across the globe [2]. WHO is determined to keep the momentum for increasing access to COVID-19 vaccines going and will continue to assist countries in accelerating vaccine delivery in order to save lives and keep people from becoming seriously ill [2]. Countries should continue to strive to vaccinate at least 70% of their populations, with a focus on vaccinating 100% of health workers, and 100% of the most vulnerable groups, such as people over 60 and those who are immunocompromised or have underlying health conditions [1]. The modus operandi to reduce the transmission therefore, was mainly behavioral such as social distancing, hand sanitization, regular washing of the hands, and wearing of face masks [3]. The COVID-19 virus is different from its predecessors, the beta coronaviruses group which includes the Middle East Respiratory Syndrome (MERS-CoV) and Severe Acute Respiratory Syndrome (SARS-CoV) as it is more easily transmitted and also more highly contagious than the other two [3].

The first case of COVID-19 in the United Arab Emirates (UAE) was detected on the January 29, 2020 involving a 73-year-old Chinese woman. The first two deaths were confirmed on the March 20, 2020 [3, 4]. A curfew was imposed four days later on the March 24, 2020 along with a nationwide disinfection strategy. The UAE government imposed a partial lockdown along with placement of testing centers all over the country. The aggressive testing approach coupled with timely medical intervention and prevention in the general population brought down the figures considerably. As of October 23, 2020 during the data collection, the number of active cases was 120,710 and 474 deaths and currently in April 2023, the number of daily new cases is approximately 90,912 and 674 deaths [4].

COVID-19 infection is characterized by high fever and cough, and in advanced cases, patients may develop respiratory distress. Other symptoms such as nausea, vomiting, diarrhea, muscle-joint pain, and loss of appetite have also been reported [5, 6]. As the understanding of COVID-19 disease process was relatively in its infancy in 2020, delivery of treatment and more so, the understanding of the disease among the general public, health care workers and researchers have jeopardized timely medical intervention and have put patients' lives at risk [7, 8]. Moreover, currently large amount of information has been misleading the general public about the disease and this has spread via social media and person-to-person contact. Misconceptions and the false knowledge have led to diminished the understanding of the disease among the health students globally [9, 10].

The knowledge and perception of these students will play an important role in how well their emergency preparedness will be in unprecedented times such as these. Gathering information related to both these factors among the students will help in raising awareness and also augment better clinical judgement and address risk perception. Furthermore, knowledge and perception studies provide data that can help overcome poor knowledge about the disease and also institute COVID-19 awareness programs [11].

Dental students need adequate training and sufficient knowledge of COVID-19 infection, prevention, and control. This requires an understanding of current guidelines concerning the disease. To date, only limited data have been collected from dental students regarding COVID-19 infection in UAE, hence this study aims to assess knowledge, attitude, and practice regarding COVID-19 among dental college students and internship students in Ajman University (AU), UAE. We believe that, the findings of this study will help health authorities to formulate policies as needed, which will be instrumental in planning awareness campaigns that can contain the pandemic effectively.

## 2. Material and Methods

A cross-sectional observational study was conducted at the College of Dentistry, AU, Ajman, UAE. The study was introduced after obtaining approval from the Research and Ethical committee of the University with reference number: D-H-F-2020-04-27. All the participants were informed about the objectives of the study and an informed consent was obtained from each student before enrollment in the study.

The study population included all the dental students in years 1–5, as well as internship students at the College of Dentistry in AU. All 900 registered dental students were contacted via emails and phone number to take part in the study.

### 2.1. Sample Size Calculation

In the current study, population size were used to determine the minimum sample size to obtain reliable statistical information to draw inferences about the whole population.

Sample size (*n*) was calculated via online Open Epi link (a collaborative, Open-Source Project in Epidemiologic Computing version 3.01) using Kish formula for sample size estimation choosing 95% significance level, a 5% margin of error, and 50% response rate, the initial representative sample size is 270. Adjustment of the sample size to account for the anticipated number of students who will not response and considered as the nonresponse rate (attrition rate) was done and 20% of sample size was added.

Adjusted sample size = estimated sample size/(1−*W*), where *W* is the proportion expected to withdraw. Accordingly, 270/(1–0.2) ≈ 338 students represented the minimum sample size, in the current study.

### 2.2. Inclusion Criteria Included

All male and female students with any nationality currently registered with AU Dental College, both undergraduate and postgraduate, and understanding of English or Arabic languages.

### 2.3. Exclusion Criteria Included

Students that were not currently registered with AU, unable to read and understand English or Arabic languages, and students from colleges apart from AU Dental College.

### 2.4. Study Instrument/Data Collection Tools

The self-administered well-structured questionnaire used in the study consisted of six measures: personal information which are mainly gender, age, status whether undergraduate or postgraduate students, and the year of study.

Knowledge domain evaluated the students regarding the definition, transmission, symptoms, incubation period, characteristics, treatment, and vaccination of COVID-19 using eight relevant questions. Knowledge scores ranged from 0 to 8 (total scoring was calculated for each participant) in which a value of 5 was consider as cutoff level, accordingly a level of <5 were set for poor knowledge and ≥5 for good knowledge. Moreover, one question was mentioned to the participants to rate their own level of knowledge for further comparisons if needed.

Risk perception (attitude) evaluation was achieved through six questions in which responses were verified on 5-point Likert scales. Positive responses with a score of 1 was assigned to strongly agree and agree answers, whereas neutral, strongly disagree, and disagree answers were scored zero for all the items except for item number 2 and 5, in which strongly disagree and disagree responses were considered positive and a score of 1 was given to them, while all other responses were scored 0. Hence, an attitude scale ranged from 0 to 6. A mean of total score of ≥3 was considered as positive attitude while score of lower than 3 was considered for negative attitude.

Preventive behavior was established using 11 related questions in which a positive score of 1 was given to preventive measures followed against COVID-19 by the participant, consequently behavior score ranged from 0 to 11 in which a score of 7 and higher was considered positive.

Moreover, practice section based mainly but not only on three hypothetical particular situations in relation to COVID-19 with multiple indirect questions and different response to the COVID-19 real case scenario in which the student needs to select the most appropriate response, where the proper practice score ranged from 0 to 3 according to each real case-scenario.

In last, sources of knowledge about Novel coronavirus was collected and the reliability of these sources where identified using Cronbach's *α* test.

All scores were based on the mean of the total number of the questions with different weight based on the difficulty level.

### 2.5. Statistical Analysis

The current survey involved mainly a close-ended questionnaire, which was validated using factor analysis. The internal reliability of all four tools (knowledge, attitude, behavior, and practice) of the questionnaire was assessed by calculating Cronbach's *α*.

Survey questionnaires distributed among the students were internally consistent of all used tools of the questionnaire as assessed by Cronbach's *α* coefficient test as follow: eight items to assess the knowledge score *α* = 0.799–0.801, six attitude related items *α* = 0.811–0.813, 11 items related to the behavior toward COVID-19 *α* = 0.766–0.768, three items related to practice *α* = 0.908–0.910, and 13 items in relation to source of information *α* = 0.845–0.847.

The data collected was entered and the statistical analyses were performed using SPSS 26.0 (SPSS, Inc., Chicago, Illinois, United States).

Assumption of normality was established to check the validity of the parametric test using Shapiro–Wilk test, which was significant (*p* < 0.05). Thus the null hypothesis was rejected as the data were not normally distributed. Therefore, nonparametric tests were used to estimate the difference between the selected variables (gender, age, and year of study) in relation to different parameters (knowledge, attitude, practice, and source of information).

Collected data were organized and tabulated as descriptive results, and they included the student's gender, age, and year of study; then the data were analyzed and tabulated as frequency and percentage distribution. Chi-square test (*χ*^2^) was applied to determine the association between study variables and different study questions.

Nonparametric independent-samples (Mann–Whitney *U* Test) was used to determine significant differences in the distribution of all study parameters (knowledge, attitude, and practice) across study categories (gender, age, and year of study).

The mean total score and standard deviation was tabulated for each of the measures under the current research using univariate analysis. Statistical analysis was performed on the four categories: eight knowledge-related items, six-items in relation to attitude, 11 behavior-related items, and three items for practical real COVID-19 cases related to COVID-19 quarantine protocol.

Differences between individual years were assessed using a pairwise comparison test. The mean scores were compared across age, participant's year of study, and gender. The variation in mean response scores between individual student populations was tested using the *t* test and Levene's test for equality of variances. Univariate ANOVA along with Tukey post hoc comparisons were done to determine variability between dependent variable groups: age, gender, and year of study.

Spearman's correlation and regression analysis were calculated to compare the strength of the effect of each independent variable to the dependent one.

The statistical significance (*p*-value) was set in this study at below 0.05 with 95% confidence interval.

## 3. Results

Seven hundred sixty-nine students (769/900, 85.4%) responded to the questionnaire, 38% of the participants were males, and 62% were females. Mean age was 25.2 ± 6.3 years old. Majority were undergraduate students (86.9%) and below 25 years of age (86.7%). Five hundred forty participants (70.2%) rated their knowledge level on COVID-19 disease as good or very good. Majorities (85.8%) of the study participants were aware about government COVID-19 task force (UAE) and less than half (48.1%) were following UAE press conferences (Table 1).

Based on the responses obtained from the study participants, the majority (75%) had adequate knowledge of COVID-19. But only 40.6% of the participants projected positive attitude. An overwhelming 94.5% of the participants projected positive behavior. The study participants had a mean (SD) practice score of 2.09 (0.56) and median (Q1–Q3) score of 2 (2–2) (Table 2).

Participants of age groups above 25 years had significantly higher proportion of subjects with adequate knowledge (88.2%), positive attitude (56.9%), and positive behavior (100%) compared to participants below 25 years age group (*p* = 0.009). All postgraduate students demonstrated adequate knowledge and positive behavior, whereas 71.3%–93.7% of the undergraduates had adequate knowledge and positive behavior (*p* < 0.001). Based on the year of the study, all the interns had adequate knowledge of COVID-19, whereas 71.4% of the clinical students and 69.8% of preclinical students had adequate knowledge. Knowledge and attitude status were not significantly different between the gender groups (*p* = 0.060). About 92.5% of the males and 95.8% of the females projected positive behavior and this difference in the distribution was found to be statistically significant (*p* = 0.04). Also, significant differences in the distribution was observed between the year of study according to attitude and knowledge (*p* < 0.001). Significantly higher proportion of participants (79.3%) with self-rated good/very Ggood knowledge demonstrated adequate knowledge regarding COVID-19 as compared to those with self-rated very poor/poor/average knowledge (*p* < 0.001) but there was no significant difference in the attitude (*p* = 0.200) (Table 3).

Postgraduate students had a significantly higher knowledge score (5.63 ± 0.75) compared to the undergraduate students (5.28 ± 1.29) (*p* = 0.030). No significant difference in the mean knowledge score was obtained between gender, age, and the year of study (*p* = 0.900, *p* = 0.170, and *p* = 0.270, respectively). Females had a significantly higher attitude (*p* = 0.005) and behavior (*p* < 0.001) score than males, whereas practice score was higher in males (*p* = 0.040). The attitude and behavior scores were significantly higher in participants above 25 years of age in comparison to those below 25 years of age (*p* < 0.001) (Table 4).

**Figure 1:**
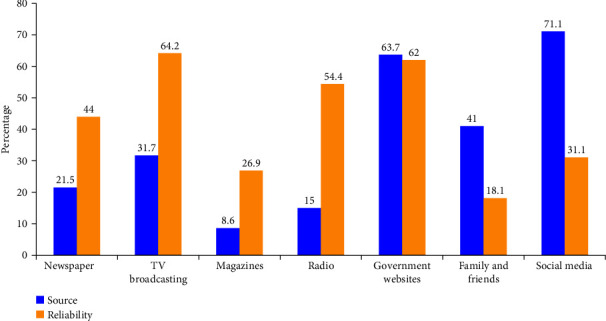
Sources of information and their reliability.

**Figure 2:**
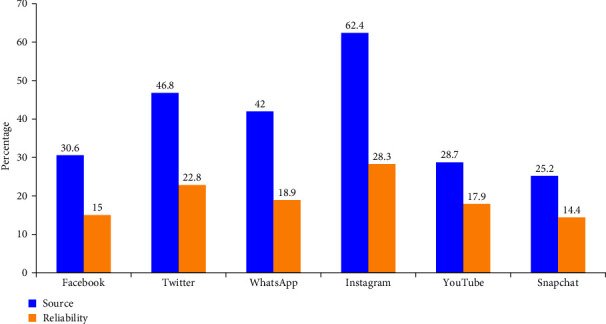
Social media sources and their reliability.

Study participants reported social media (71.1%), government websites (63.7%), and family and friends (41.0%) as the top three sources of information regarding COVID-19 related information. Government websites (62.0%) along with TV broadcasting (64.2%) are considered the most reliable sources by the participants (Figure 1). Among the social media, Instagram (62.4%), Twitter (46.8%), and WhatsApp (42.0%) were the top three sources. However, they were not considered very reliable from the participant's point of view (31.1%) (Figure 2).

## 4. Discussion

Adequate understanding of COVID-19 among healthcare workers including dental/internship students (DS) is crucial to curbing the COVID-19 outbreak as it is only with adequate levels of the knowledge that DS can comprehensively identify, diagnose, manage COVID-19 cases, and prevent the transmission of the disease.

The excellent response rate (85.4%) obtained in this study, shows the importance of mass administration of questionnaires. In comparison with other studies, the findings obtained in this analysis were favorable in terms of incidence and circumstances around COVID-19 [12, 13].

In this study, the key findings were that most DS (75%) had adequate knowledge of COVID-19. These findings are comparable to reports from other countries in which students showed excellent knowledge of the COVID-19 virus infection [12]. There were no major differences in awareness and attitude status between the gender groups (*p* > 0.05). Positive conduct was expected by 92.5% of male and 95.8% of female and this disparity in the distribution was found to be statistically important (*p* = 0.04). Participants of age groups above 25 years had significantly higher proportion of subjects with adequate knowledge (88.2%), positive attitude (56.9%), and positive behavior (100%) as compared to participants of below 25 years age group, this is perhaps a reflection of more clinical exposure and knowledge of the senior final year and postgraduate students compared with junior undergraduate students. All postgraduate students demonstrated adequate knowledge and positive behavior, whereas 71.3% and 93.7% of the undergraduates demonstrated adequate knowledge and positive behavior.

The findings of this study indicates that although adequate knowledge was observed among the AU dental students, some aspects of the COVID-19 infection was deficient such as knowledge of the incubation period of the COVID-19 virus or mode of transmission of infection. This is a cause of concern which needs the academic institutions and the health authorities concerned to step up their efforts to provide health authorities with a large number of resources to educate DS and develop their awareness of COVID-19.

Further analysis of the knowledge-related questions toward COVID-19 among dental students, revealed that 83% of DS at AU demonstrated a good knowledge of COVID-19 definition, symptoms, and the current available treatment or vaccine available for COVID-19. Likewise, poor knowledge was clearer in questions related to spread of the disease and characteristics of COVID-19. Such findings were similar to results by other researcher [14].

Our results are different from the findings of LincangoNaranjo's study from Ecuador [15], Spain [16], and Turkey [17] in which participants had insufficient awareness of the symptoms of COVID-19. It can thus be suggested that this is a result of the positive influence of the regular infection control workshops conducted by the infection control team on COVID-19 pandemic in our university. Furthermore, the findings of the Uganda awareness survey showed that the majority of the participants (88.0%) had correct knowledge of the main symptoms of COVID-19 and 92.4% of the participants were aware that early symptomatic and supportive treatment would help most of the patients to recover from the infection [18]. The WHO guidelines refer to particles >5–10 *µ*m in diameter as respiratory droplets, while airborne transmission occurs in droplet nuclei which are particles [19, 20]. In addition, most of the Uganda [18] and Iran [21] medical students were able to correctly recognize the major clinical symptoms of COVID-19. About 71.1% of DS used social media as a source of information about COVID-19, a finding of notable concern as the type and quality of information about COVID-19 in the social media is a cause of concern as highlighted by many authorities [22–24] as during a time of social distance and limited contact with others, social media became an important place to interact during the COVID-19 pandemic. Social media platforms helped the world remain connected, largely increasing in usage [19].

Among the social media, Instagram (62.4%), Twitter (46.8%), and WhatsApp (42.0%) are the top three sources. However, they are not considered very much reliable by the participants (31.1%). In addition, more than half of the participants believed that TV broadcasting (64.2%), and government websites (62 or 63%%) are the most reliable sources of information. Similar reports from the different centers confirm that social media is the major source of information followed by television [14, 19, 25]. Although, it was reported that social media as a source of information is cost-effective and easily accessible, but could also spread fake information faster, which can have devastating effects on the society [26]. In this study, we found that more than 63% of DS used official government websites as a main source of COVID-19 knowledge. This indicates that the COVID-19-related updates released by official government health authorities online have had a significant effect on improving the level of DS awareness. This is encouraging because the use of authentic sources for COVID-19 information and notifications is a key factor in providing students with transparent information and is important for DS preparedness and response. Furthermore, DS should be familiar with and carefully evaluate all coronavirus related websites and any awareness materials before sharing or applying it to avoid misinformation [27, 28].

In this study, 40.6% of Ajman DS showed positive attitude toward COVID-19 pandemic which is lower than the findings reported from medical students in India (73.2%) [29], Uganda (94%) [18], and Iran (93.8%) [21].

In the current study, nearly all of the DS responded positively about the preventive measures to control spread of the virus such as home quarantine (97.6%), washing hands before and after any task (95%), wearing facemasks (85%), avoiding people with fever (93.6%), and avoiding using public transportation (95.1%). Further analysis revealed that most of the preventive behavior-related questions showed no significant difference between gender or study level (*p* > 0.05). A similar study also reported among participants in a survey among the government medical college in India, in which 96.9% of students agreed that COVID-19 could be prevented by adapting precautionary measures such as avoiding public transportation [29]. Jordanian medical students suggested that the most effective techniques for mitigating COVID-19 infection were personal hygiene and quarantine [19].

Most of the students reacted positively to the practice-based scenarios, with only 26.1% positive response to allow children of medical staffs working at COVID-19 hospitals to be present with relatives or children of their family are in same class.

The questionnaire's case-based scenarios and lack of many direct questions may have contributed to the poor scores found in this study. However, the simulation might better reflect the participants' intentions. The use of case-based situations in this study may be a superior approach to successfully highlighting the important gaps and correlations.

Comprehensive evaluation of the practical situations related to real COVID-19 case-scenario revealed a higher score of females than males with no significant difference in relation to gender, this is comparable to results of study from India [29], Bangladesh [30], and China [31, 32].

Various dental procedures were reported to cause aerosol spread of viruses [33], others occur during preclinical laboratory exercises or in a dental laboratory. COVID-19 virus transmission can also occur with droplets ejected during speaking, coughing, or sneezing [29]. Therefore, to eliminate the risk of transmission of viral infections in clinical settings, avoidance of aerosol exposure remains key. Dental colleges must invest resources in educating students and supporting team on the correct use of instruments and adopting effective cross infection protocols when interacting with patients in order to reduce their possible risk of exposure to the COVID-19 infection via aerosol spread. If these steps are reinforced in the dental school, they have a better chance of being followed once the student enters private practice. Several studies have shown that insufficient awareness and compliance of the students during the pandemic [34, 35]. DS are particularly susceptible to unintentional exposure to potentially contaminated material because they lack the experience and capacity to perform dental procedures [36]. In addition, the COVID-19 virus has been found in infected patients' saliva, therefore, DS and other healthcare professionals in particular should be very vigilant in shielding themselves from transmission of the disease during clinical procedures [37, 38].

Previous studies have shown that their readiness to treat patients with infectious diseases increases as the level of awareness of DS increases [39, 40].

In this regard, it is promising that the results of the study showed that dental students at AU expressed hope that COVID-19 will be regulated because of the rapid spread and rise in the mortality rate associated with COVID-19 globally, governments and public health professionals focused their attention on precautionary safety measures at both individual and community levels.

Many precautionary measures have been implemented to protect patients when visiting dental clinics, such as the use of masks, temperature screening, and hand hygiene at the clinic. Individuals are aware of the precautions that should be taken, and similar findings can be found in the literature [41].

The American Environmental Protection Agency (EPA) currently recognizes copper as the best antimicrobial metal. The EPA has even approved the registration of copper alloys as “antimicrobial materials with public health benefits,” allowing manufacturers to make legal claims about the public health benefits of registered alloy-based products. Bedrails, handrails, over-bed tables, sinks, faucets, doorknobs, toilet hardware, computer keyboards, health-club equipment, and shopping cart handles are among the antimicrobial copper products approved by the agency [42].

Another important finding of this research was that most of the DS had a good outlook toward COVID-19. Moreover, 97.6% of the students agreed to quarantine themselves if needed, to control the spread of the infection. These results are likely to be related to a lack of adequate knowledge by some of the DS about the current and important prevention and isolation strategies.

The searches for “vaccine” have reached an all-time high globally, and according to the WHO, at least 198 COVID-19 vaccines are undergoing development, with 44 currently being clinically evaluated [43]. A safe and effective anti-COVID-19 vaccine would go a long way toward helping society return to its prepandemic normal.

Considering that the present study assessed only limited demographic variables, it is recommended that more demographic factors like different age groups, more diverse population from different sectors should be included in further studies.

## 5. Conclusion

The results of this study show that AU dental and internship students have adequate knowledge and positive attitude of the COVID-19 pandemic but the majority are not eager to adopt effective strategies to avoid the spread of COVID-19. Further education should be offered to encourage efficient infection management practices to protect students, faculty, and other university staff.

## Figures and Tables

**Table 1:** Demographic details, knowledge, attitude, behavior, and practice among the study participants.

			*N* (%)
Gender	Male		292 (38.0)
	Female		477 (62.0)

Age	<25 years		667 (86.7)
	>25 years		102 (13.3)

Status	Undergraduate		668 (86.9)
	Postgraduate		101 (13.1)

Year of study	Preclinical (*n* = 275)	First year	118 (15.3)
		Second year	157 (20.4)
	Clinical (*n* = 381)	Third year	126 (16.4)
		Fourth year	118 (15.3)
		Final year	137 (17.8)
	Internship (*n* = 113)		113 (14.7)

How would you rate your knowledge level on COVID-19 disease?	Very poor		3 (0.4)
	Poor		4 (0.5)
	Average		222 (28.9)
	Good		387 (50.3)
	Very good		153 (19.9)

Awareness about government COVID-19 task force (UAE)	No		109 (14.2)
	Yes		660 (85.8)

Following UAE press conferences	Always		370 (48.1)
	More often		11 (1.4)
	Never		93 (12.1)
	Sometimes		295 (38.4)

Knowledge	Inadequate		192 (25.0)
	Adequate		577 (75.0)

Attitude	Negative		457 (59.4)
	Positive		312 (40.6)

Behavior	Negative		42 (5.5)
	Positive		727 (94.5)

Practice score	Mean (SD)		2.09 (0.56)
	Median (Q1–Q3)		2 (2–2)

**Table 2:** Responses to knowledge, attitude, behavior, and practice questions.

		*N* (%)
Knowledge (correct response)	Which of the following is correct about the definition of COVID-19?	653 (84.9)
	Which of the following is correct about transmission route of COVID-19?	669 (87.0)
	Which of the following is NOT correct about “close contact” of COVID-19?	362 (47.1)
	Which of the following is correct about symptom of COVID-19?	733 (95.3)
	Which of the following is correct about the incubation period of COVID-19?	383 (49.8)
	Which of the following explanations is NOT correct about the characteristics of COVID-19?	209 (27.2)
	Which one is incorrect about self-isolation of COVID-19?	398 (51.8)
	Which one is correct about treatment or vaccine of COVID-19?	691 (89.9)

Attitude (response indicating positive attitude)	I think that I will contract COVID-19 if I come into contact with a COVID-19 patient	565 (73.5)
	I think that I might contract COVID-19 even if I do not come into contact with a COVID-19 patient	363 (47.2)
	My health will be severely damaged if I contract COVID-19	253 (32.9)
	I think COVID-19 is more severe than any other respiratory diseases	247 (32.1)
	Even if I fall ill with another disease, I will not go to hospital because of COVID-19	374 (48.6)
	COVID-19 will inflict serious damage to my community	623 (81.0)

Behavior (response indicating positive behavior)	If possible, I don't leave home	746 (97.0)
	I washed my hands with water and soap before and after I left home	728 (94.7)
	I didn't touch my ears, nose, and/or mouth with hands I had not washed	678 (88.2)
	I avoided contact with people who had fever or respiratory symptoms	720 (93.6)
	I washed my hands with hand sanitizers when I was outside	724 (94.1)
	I wore a mask when I go outside	654 (85.0)
	I avoided crowded places	723 (94.0)
	I go to hospital much less	696 (90.5)
	I avoided using public transportation	731 (95.1)
	I ate food that would strengthen my immune system	571 (74.3)
	I exercised to strengthen my immune system	493 (64.1)

Practice (response indicating good practice)	Usually you will try to help COVID-19 patient by yourself	295 (38.3)
	Do you think you could sacrifice yourself when a national-wide disaster or crisis occurs?	553 (71.9)
	How would you react if you are suspected of being a COVID-19 carrier and are recommended to be in self-isolation?	704 (91.5)
	How do you usually react when an emergency situation or crisis arises	389 (50.6)
	How would you react if you realize your relatives or children of your family are in the same class with children of someone suspected (or confirmed) of having COVID-19?	700 (91.0)
	How would you react if you realize my relatives or children of my family are in same class with children of medical staffs working at COVID-19 hospitals? (Including school, extracurricular activities)	201 (26.1)

**Table 3:** Comparison of knowledge, attitude, and behavior according to gender, age, year of study, and self-rated knowledge.

		No.	Knowledge		Attitude		Behavior	
			Inadequate	Adequate	Negative	Positive	Negative	Positive
Gender	Male	292	84 (28.8%)	208 (71.2%)	186 (63.7%)	106 (36.3%)	22 (7.5%)	270 (92.5%)
	Female	477	108 (22.6%)	369 (77.4%)	271 (56.8%)	206 (43.2%)	20 (4.2%)	457 (95.8%)
	*χ* ^2^ test		*χ* ^2^ value = 3.63, *p* = 0.06 (NS)		*χ* ^2^ value = 3.56, *p* = 0.06 (NS)		*χ* ^2^ value = 3.92, *p* = 0.04 ^*∗*^	

Age	<25 years	667	180 (27.0%)	487 (73.0%)	413 (61.9%)	254 (38.1%)	42 (6.3%)	625 (93.7%)
	>25 years	102	12 (11.8%)	90 (88.2%)	44 (43.1%)	58 (56.9%)	0 (0.0%)	102 (100.0%)
	*χ* ^2^ test		*χ* ^2^ value = 10.94, *p* = 0.001 ^*∗*^		*χ* ^2^ value = 12.94, *p* < 0.001 ^*∗*^		*χ* ^2^ value = 6.79, *p* = 0.009 ^*∗*^	

Status	Undergraduate	668	192 (28.7%)	476 (71.3%)	405 (60.6%)	263 (39.4%)	42 (6.3%)	626 (93.7%)
	Postgraduate	101	0 (0.0%)	101 (100.0%)	52 (51.5%)	49 (48.5%)	0 (0.0%)	101 (100.0%)
	*χ* ^2^ test		*χ* ^2^ value = 38.69, *p* < 0.001 ^*∗*^		*χ* ^2^ value = 3.04, *p* = 0.08 (NS)		*χ* ^2^ value = 6.72, *p* = 0.01 ^*∗*^	

Year of study	Preclinical students	275	83 (30.2%)	192 (69.8%)	144 (52.4%)	131 (47.6%)	17 (6.2%)	258 (93.8%)
	Clinical students	381	109 (28.6%)	272 (71.4%)	265 (69.6%)	116 (30.4%)	25 (6.6%)	356 (93.4%)
	Interns	113	0 (0.0%)	113 (100.0%)	48 (42.5%)	65 (57.5%)	0 (0.0%)	113 (100.0%)
	*χ* ^2^ test		*χ* ^2^ value = 44.29, *p* < 0.001 ^*∗*^		*χ* ^2^ value = 35.36, *p* < 0.001 ^*∗*^		*χ* ^2^ value = 7.70, *p* = 0.02 ^*∗*^	

Self-rated knowledge level on COVID-19 disease?	Very poor/poor/average	229	80 (34.9%)	149 (65.1%)	144 (62.9%)	85 (37.1%)	6 (2.6%)	223 (97.4%)
	Good/very good	540	112 (20.7%)	428 (79.3%)	313 (58.0%)	227 (42.0%)	36 (6.7%)	504 (93.3%)
	*χ* ^2^ test		*χ* ^2^ value = 17.29, *p* < 0.001 ^*∗*^		*χ* ^2^ value = 1.61, *p* = 0.20 (NS)		*χ* ^2^ value = 5.10, *p* = 0.02 ^*∗*^	

^*∗*^Statistically significant. NS, nonsignificant.

**Table 4:** Comparison of knowledge, attitude, behavior, and practice score according to gender, age, year of study, and self-rated knowledge.

Gender			No.	Mean (SD)	Range	Median (Q1–Q3)	*P*-value
Knowledge	Gender	Male	292	5.30 (1.31)	0–8	6 (4–6)	0.90 (NS)^#^
		Female	477	5.35 (1.19)	2–8	5 (5–6)	
	Age	<25 years	667	5.34 (1.27)	0–8	6 (4–6)	0.17 (NS)^#^
		>25 years	102	5.25 (1.00)	2–7	5 (5–6)	
	Status	UG	668	5.28 (1.29)	0–8	5 (4–6)	0.03^*∗*^^,#^
		PG	101	5.63 (0.75)	5–7	5 (5–6)	
	Year of study	Preclinical	275	5.33 (1.37)	0–8	6 (4–6)	0.27 (NS)^##^
		Clinical	381	5.25 (1.25)	2–7	5 (4–6)	
		Intern	113	5.57 (0.73)	5–7	5 (5–6)	

Attitude	Gender	Male	292	20.76 (2.95)	14–27	21 (18–23)	0.005^*∗*^^,#^
		Female	477	21.38 (3.04)	12–30	22 (20–23.5)	
	Age	<25 years	667	21.03 (3.08)	12–30	21 (19–23)	0.001^*∗*^^,#^
		>25 years	102	21.88 (2.45)	16–25	22 (22–23)	
	Status	UG	668	21.18 (3.12)	12–30	21 (19–23)	0.45 (NS)^#^
		PG	101	20.94 (2.24)	18–24	22 (18–22)	
	Year of study	Preclinical	275	21.49 (3.17)^a^	12–30	22 (19–24)	0.003^*∗*^^,##^
		Clinical	381	20.77 (3.00)^a,b^	14–28	21 (19–23)	
		Intern	113	21.58 (2.50)^b^	18–25	22 (18–24)	

Behavior	Gender	Male	292	9.18 (2.27)	0–11	10 (9–11)	<*0.001*^*∗*^^,#^
		Female	477	10.03 (1.45)	5–11	11 (10–11)	
	Age	<25 years	667	9.62 (1.93)	0–11	10 (9–11)	0.001^*∗*^^,#^
		>25 years	102	10.27 (1.03)	8–11	11 (9–11)	
	Status	UG	668	9.63 (1.94)	0–11	10 (9–11)	0.07 (NS)^#^
		PG	101	10.20 (0.89)	9–11	11 (9–11)	
	Year of study	Preclinical	275	9.56 (2.28)^a^	0–11	11 (9–11)	0.009^*∗*^^,##^
		Clinical	381	9.64 (1.68)^b^	5–11	10 (9–11)	
		Intern	113	10.28 (0.88)^a,b^	9–11	11 (9–11)	

Practice	Gender	Male	292	2.13 (0.67)	0–3	2 (2–3)	0.04^*∗*^^,#^
		Female	477	2.06 (0.48)	0–3	2 (2–2)	
	Age	<25 years	667	2.08 (0.57)	0–3	2 (2–2)	0.18 (NS)^#^
		>25 years	102	2.17 (0.47)	1–3	2 (2–2)	
	Status	UG	668	2.05 (0.56)	0–3	2 (2–2)	<*0.001*^*∗*^^,#^
		PG	101	2.35 (0.48)	2–3	2 (2–3)	
	Year of study	Preclinical	275	2.06 (0.60)^a^	0–3	2 (2–2)	0.001^*∗*^^,##^
		Clinical	381	2.05 (0.55)^b^	0–3	2 (2–2)	
		Intern	113	2.27 (0.45)^a,b^	2–3	2 (2–3)	

^#^Mann–Whitney *U* test. ^##^Kruskal–Wallis Test.  ^*∗*^*p* < 0.05 Statistically significant, *p* > 0.05. NS, nonsignificant. Pairwise comparison using Mann–Whitney *U* test statistically significant for pairs with similar superscript – ^a,b^.

## Data Availability

Data supporting this research article are available from the corresponding author or first author on reasonable request.

## Additional Points

*Limitations*. Given that this was a cross-sectional study and that the data presented depend on the integrity and recall of the students, there is a possibility that some students will overestimate or underestimate while responding to the questions. Furthermore, online surveys and web-based research, can be subject to considerable bias. In addition, external validity is challenging as the results from our study cannot be generalized to other populations. In view of these limitations, to examine the understanding, attitude and practice of COVID-19 in different countries, further studies with a broad sample size should be carried out along with strengthening the research design taking into consideration the risk of bias, presence of confounding, using more randomized sampling, and appropriate study design.
